# Lost in Transition: Health Care Experiences of Adults Born Very Preterm—A Qualitative Approach

**DOI:** 10.3389/fpubh.2020.605149

**Published:** 2020-11-30

**Authors:** Anna Perez, Luise Thiede, Daniel Lüdecke, Chinedu Ulrich Ebenebe, Olaf von dem Knesebeck, Dominique Singer

**Affiliations:** ^1^Section Neonatology and Pediatric Intensive Care Medicine, Center for Obstetrics and Pediatrics, University Medical Center Hamburg-Eppendorf, Hamburg, Germany; ^2^Center for Psychosocial Medicine, Institute of Medical Sociology, University Medical Center Hamburg-Eppendorf, Hamburg, Germany

**Keywords:** adults born preterm, longterm effects of prematurity, self-reported health, health care needs, health care satisfaction, barriers to health care, qualitative research

## Abstract

**Introduction:** Adults Born Very Preterm (ABP) are an underperceived but steadily increasing patient population. It has been shown that they face multiple physical, mental and emotional health problems as they age. Very little is known about their specific health care needs beyond childhood and adolescence. This article focuses on their personal perspectives: it explores how they feel embedded in established health care structures and points to health care-related barriers they face.

**Methods:** We conducted 20 individual in-depth interviews with adults born preterm aged 20–54 years with a gestational age (GA) below 33 weeks at birth and birth weights ranging from 870–1,950 g. Qualitative content analysis of the narrative interview data was conducted to identify themes related to self-perceived health, health care satisfaction, and social well-being.

**Results:** The majority (85%) of the study participants reported that their former prematurity is still of concern in their everyday lives as adults. The prevalence of self-reported physical (65%) and mental (45%) long-term sequelae of prematurity was high. Most participants expressed dissatisfaction with health care services regarding their former prematurity. Lack of consideration for their prematurity status by adult health care providers and the invisibility of the often subtle impairments they face were named as main barriers to receiving adequate health care. Age and burden of disease were important factors influencing participants' perception of their own health and their health care satisfaction. All participants expressed great interest in the provision of specialized, custom-tailored health-care services, taking the individual history of prematurity into account.

**Discussion:** Adults born preterm are a patient population underperceived by the health care system. Longterm effects of very preterm birth, affecting various domains of life, may become a substantial burden of disease in a subgroup of formerly preterm individuals and should therefore be taken into consideration by adult health care providers.

## Introduction

Nowadays 5–13% of all infants are born preterm, i.e., at a **g**estational **a**ge (GA) of <37 weeks. Very small preterm infants with a GA of <32 weeks and/or a birthweight <1,500 g comprise about 1,5% of all live births per year (1, 2).

Due to medical and technical advances in neonatology over the last decades, survival rates of very preterm infants have substantially increased. Since the 1980s, when the causal treatment of lung immaturity by intratracheal surfactant application became widely available, the majority of pre-mature infants, down to the very immature babies born at the threshold of viability, survive (3, 4).

As a consequence, an increasing number of formerly very preterm infants is growing up, leaving infancy, childhood and adolescence and thus, the professional competency of the pediatric health care provider, behind. Not only do most very low birthweight (VLBW, birthweigth <1,500 g) and extremely low birthweight (ELBW, birthweigth <1,000 g) preterm infants since the 1980s reach adulthood. This fairly new and constantly increasing population of adults born preterm (ABP) is facing a new and–before the era of intratracheal surfactant application rarely existent–challenge: they age.

It is known that ABP are at increased risk for developing long-term sequelae at various organ sites including cardio-vascular, pulmonary, metabolic, renal, visual, obstetric, and psychiatric impairments (1, 2, 5–18).

Severe neurologic impairments such as cerebral palsy and hydrocephalus still affect about 2–9% of all very small preterms today. (2) More subtle neuro-cognitive, behavioral and socio-emotional dysfunctions have been a major focus of research over the last decades, also affecting 1/4–1/3 of all very small preterm survivors (19–22).

Another important aspect when looking at the long-term trajectories of very preterm birth is that ex-preterms seem to have a higher risk for age-associated diseases, such as cardiovascular disease. This implies that age-associated changes and morbidities may occur earlier and more frequently in ABP compared to adults born at term (11, 23). In this context, one discussed mechanism is the “fetal origin hypothesis,” which states that increased risk is programmed during fetal life. Adverse effects during pregnancy, such as intrauterine growth retardation followed by several weeks of unphysiologic extrauterine maturation of VLBW and ELBW infants lead to metabolic reprogramming which may cause diseases in later adulthood (24). Subjects born preterm, who have a low birthweight and more stress during fetal and early life, could be programmed to a different health outcome in later life (25).

When considering the available data it becomes clear that prematurity is a burden of potentially lifelong character.

To date, very little is known about ABP's health care needs beyond childhood and adolescence (26). After they reach adulthood and transition from pediatric into adult health care, there are no more custom-tailored health care services to address their needs. We hypothesized that their status as “formerly born very preterm” is largely underperceived among adult health care providers and may therefore not be integrated into counseling, prevention, or treatment concepts.

In this pilot study, a qualitative approach is applied to address the following research questions: (1) How do ABP perceive their health status as adults? (2) What are ABP's experiences with health care, in particular how do ABP feel perceived by established health care structures and services and what barriers to health care are they facing?

## Materials and Methods

In this qualitative study semi-structured in-depth interviews with adults formerly born very preterm were conducted.

The rationale for choosing a qualitative research approach was the aim to assess participants' personal perspectives toward health and healthcare access in contrast to quantitative approaches that focus on objectively measurable health outcomes but may provide a less detailed insight into the situation of those formerly born preterm. To date there is a paucity of information on the personal perspectives of adults formerly born preterm with regard to their own quality of life, their fears and their hopes as they age (27). Qualitative research is particularly suitable for exploratory work where the researcher may not be aware of all the relevant aspects of the study subject in advance. Qualitative methods of data collection allow an open approach to the investigated phenomena and enable the researcher to receive a more concrete, more plastic picture from the perspective of those affected (28).

Data collection lasted 3 months from November 2018 to February 2019.

Participants were mainly recruited via the websites and contacts of two patient and parent advocacy groups, one of them being active on a regional (city of Hamburg and surroundings), the other one on a national level. Eight participants were recruited at an event for ABP organized by the national advocacy group. The remaining 12 participants became aware of the study through independent internet research or randomly volunteered to participate in the study, of which they had heard by word of mouth.

Eligible participants were >20 years of age and born with <33 weeks of gestation and/or a birthweight of <1,500 g. None of the patients had attended any kind of systematic long-term follow-up program regarding their prematurity. In- and exclusion criteria can been seen in Table 1. Participants were sampled purposefully on an entirely voluntary basis. Written consent, in which anonymity and confidentiality was granted, was obtained from all study participants. Ethical approval was granted by the ethical committee of the Hamburg Medical Association.

**Table 1:** In- and exclusion criteria of study participants.

**Inclusion criteria**	**Exclusion criteria**
Very preterm birth <33 GA ^*^	Participation in any kind of long term follow up program regarding preterm birth
Very low birth weight <1,500 g^*^	Birth and childhood abroad
Current age >20 years	Limited german language skills
German health insurance	Foreign residence
Availability of information on GA and birth weight	

* *One of the two inclusion criteria had to be fulfilled*.

Before the personal interview, each participant filled out a short written questionnaire. The short questionnaire included demographic information, perinatal facts, present diagnoses, level of education, questions about today's relevance of former prematurity and questions on current health care.

All participants completed an audiotaped, 30–60-min semi-structured interview. The interviews were conducted personally either in-person or by video call by a female medical student with a Bachelor's degree, who was trained by two of the co-authors to conduct the interviews. Interview participants knew the interviewers name and the purpose of the study. After 20 conducted interviews, thematic saturation was reached as no new themes emerged from the data (29). According to simulation studies, for a sample size like ours (*n* = 20) the probability of encountering new topics or codes is lower than 15% (30). Interviews were transcribed verbatim, following published transcription rules (31). They were repeatedly read by the two first authors and compared to the audio recordings. Each interview was read and coded by one author and then re-evaluated by the other another author. In this way, an interpretative consensus was reached. This method was preferred to parallel independent coding using statistical measures of interrater reliability, such as kappa statistics, because the small number of interviews allowed for a more comprehensive double evaluation.

The semi-structured interview guide was developed by review of the literature and expert opinion and was iteratively refined during the data collection period. The final guide is available upon request.

We performed a qualitative content analysis on transcribed interviews. (32) In a content analysis approach, the researcher seeks to gain a deep understanding of the concepts involved in the subject, and words and sentences are categorized into main categories and subcategories that make the phenomenon easier to understand. (33)

Authors systematically coded participants' responses individually and then as a group to classify codes in common themes. The coded texts were labeled with both deductive and inductive codes. The deductive codes were derived from the interview guide and the inductive codes were developed iteratively by reading and re-reading the transcribed interviews. The final code system contained 11 deductive codes that represent the interviews' main categories: role of prematurity in today's everyday life, physical health status, mental health status, health care, education and professional life, family, romantic relationships/partnerships, and leisure time activities. The applied subcodes allowed a more detailed exploration of the interview content and enabled the recognition of parallels and connections within the individual stories and perspectives of the participants.

Cross tables were used to widen analysis in order to understand differences in self-perceived health and health care seeking behavior related to former prematurity.

Herefore, different subcodes were combined with each other and with biometric data and then contrasted. This way, contrasting comparisons between different groups within the sample of ABP were made.

The software MAXQDA was used to facilitate analysis of the coded transcripts.

Representative quotations were selected to illustrate key themes.

## Results

A total of 20 adults formerly born preterm (ABP) were recruited and interviewed (Table 2). More than half of the participants were female (60%) and all of them were Caucasian (100%). Their age ranged from 20 to 54 years, the majority of participants being in the third decade of life, between 20 and 29 years of age (55%). GA at birth was between 26 and 33 weeks with a median of 29 weeks. Birthweight was between 870 g and 1,950 g with a median of 1,140 g. The sample did not include participants born small for gestational age (SGA), i.e., with a birthweight below the 10th growth percentile. There was an overrepresentation of high educational levels with the majority of participants holding a high school diploma corresponding to University entrance level, e.g., the German Abitur or Fachabitur (70%) compared to the general German population (34, 35). The study sample comprised ABP with and without physical disabilities. None of the participants had attended any kind of systematic long-term follow-up program regarding their prematurity.

**Table 2:** Characteristics of study participants.

**Characteristics**	**Participants absolute numbers (*n* = 20)**	**Participants %**
**Sex**		
Female	12	60
Male	8	40
**Age**		
Median 31,7 yrs.		
20–29	11	55
30–39	5	25
40–49	2	10
50–59	2	10
**Birthweight**		
Median 1,140 g		
<1,000 g	5	25
1,000–1,500 g	11	55
>1,500 g	4	20
**Gestational Age at Birth (GA)**		
Median 28 weeks		
Extremely preterm (<27 weeks)	4	20
Very preterm (27–32 weeks)	13	65
Preterm (>32 weeks)	3	15
**Education**		
High school diploma	14	70
Secondary school diploma	3	15
Lower secondary school diploma	1	5
Vocational school diploma	1	5
No higher-level school diploma	1	5

Codes and subcodes were established according to the data analysis description in the methods section. A visualization of the final code system is presented in the Supplementary Material: Analytic code-system.

### Role of Prematurity in Today's Everyday Life

Most participants (85%) reported that their former prematurity was still of concern in their everyday life as adults. These concerns included consequences of physical disability, mental health sequelae, difficulties in social/attachment behavior, and barriers in achieving their educational and professional goals.

### Physical Health Status

More than half (65%) of the participants reported chronic clinical diagnoses which they had received from their health care providers (Table 3). In addition to clinical diagnoses the following themes concerning physical health and fitness in everyday life were repeatedly named: low basal energy level, low physical performance, fast exhaustibility, and increased need for rest (Table 4).

**Table 3:** Physical health diagnoses.

**Diagnosis**	**Participants absolute numbers (*n* = 20)**	**Participants %**
Cerebral palsy with spasticity	5	25
Neurogenic bladder and intestinal dysfunction	1	5
Hydrocephalus with shunt system	1	5
Chronic pain syndrome	1	5
Myopia	3	15
Strabism	3	15
Amblyopia	1	5
Hearing loss	2	10
Tinnitus	1	5
Chronic back pain	1	5
Immunodeficiency	1	5
Migraine	1	5
Asthma	1	5
Stuttering	2	10
Chronic urticaria	1	5
Grave's disease	1	5

**Table 4:** Self-perceived physical health.

**Self-percieved physical health**	**Participants absolute numbers (*n* = 20)**	**Participants %**
Decreased physical performance capacity	7	35
Low basal energy level	6	30
High susceptibility to disease	5	25
Sleep disorder	3	15
Lack of body awareness	2	10

Participants referred to a “weak immune system” by which they meant high disease susceptibility and long recovery periods after acute illness.

Current age seems to influence the perception of long-term effects of former prematurity. Physical health sequelae attributed to former prematurity were more often perceived among adults born preterm <30 years of age (67%) and less frequently among adults born preterm >30 years of age (33%). In our sample, the prevalence of physical impairments was higher in the group of younger ABP. All ABP with cerebral palsy (*n* = 5) were under the age of 34.

“*I always feel as if I'm constantly at my limits. And others, they are fit, they go out in the evenings, they do this and do that. I have to constantly balance my energy, constantly.”* (Participant 5, age 53)

“*And I'm constantly sick. Really, like a weakling, who always has something. And I never feel really fit, I always catch something.”* (Participant 16, age 26).

### Mental Health Status

Almost half of the participants (45%) reported clinical diagnoses which they had received from their health care providers (Table 5). 40% of participants reported that they were currently undergoing psychotherapy, 15% said that they had undergone psychotherapy in the past, 10% said they were currently contemplating if they should undergo psychotherapy. Only 5 participants (25%) reported no affiliation to psychotherapy.

**Table 5:** Mental health diagnoses.

**Diagnosis**	**Participants absolute numbers (*n* = 20)**	**Participants %**
Depression	5	25
Regulation disorder	2	10
Anxiety disorder	1	5
Burnout	1	5
Trauma	1	5

In addition to clinical mental health diagnoses the following difficulties in everyday life were repeatedly named:

a) overstimulation/hypersensitivity, i.e., difficulty in filtering out stimuli from the surroundings (25%)
b) anxiety, especially concerning separation/loss in social life and fear of failure in private and professional life (25%)
c) awareness of own cognitive constraint (25%)
d) high sensitivity to stress (25%)

a) “*I have extreme problems in filtering out auditive stimuli from the surroundings. For example going to the supermarket is hell for me, it is only possible with my earphones on, otherwise it's not possible.”* (Participant 16, age 26 years old)

b) “*…I had, for example at school age, especially in the first years, extreme loss and separation fears. So, I could not stay at school by myself, they (the parents) had to stay with me for some time before they could leave. (.) this fear of loss or this separation fear has been present for me throughout the years.”* (Participant 2, age 31)

c) “*So, I wouldn't say that I'm 28 years old by my developmental status. I also notice that my age is very often not correctly estimated, mostly people judge me as being 18, 19 or maximal 20 years old.”* (Participant 3, age 28)

d) “*Well, stress is an unbelievable burden to me. Much more, I think, than it is for other people. I somehow also need my own rhythm. (…) and too many things assailing me, that really exhausts me. More than (it exhausts) others, I believe.”* (Participant 5, age 53)

Overall, half of all interviewed ABP stated that they were certain, that their pre-mature birth had an influence on their mental health in adult life and another 25% stated, that they assumed an influence of their prematurity on their mental health, but were not sure about causalities. Post-natal separation from the mother was named as one assumed cause affecting mental health in adult life.

“*I do see a correlation with this having been born too early, with this not being together with or inside my mother for the regular amount of time. I somehow see a correlation (with my mental health today), for me personally, yes.”* (Participant 2, age 31)

“*I do believe that a certain amount of hypersensitivity could be a possible aftereffect (of my prematurity). Um, because yes, I know that now only intellectually, when one was exposed to the environment as a preterm, the light and the sounds and one was just not protected anymore, that is why today I just have a stronger sensitivity.”* (Participant 19, age 43)

“*I need very much physical contact and I know for sure that this is also related to my pre-mature birth. (…) I was in the incubator for three months and at that time there was no possibility to have much physical contact to my mother and so I definitely know that this comes from my preterm birth.”* (Participant 4, age 31)

Among ABP <30 years of age mental health sequelae which are attributed to prematurity were less perceived than among ABP >30 years of age. In ABP >40 years of age *all* participants reported mental health sequelae which they attributed to their former prematurity, whereas in the age group of 20–29 years of age mental health sequelae were reported in only 36%.

“*(…) I sometimes have something like an emotional backflash and I feel things of which I rationally think that they have nothing to do with the here and now, but that these are feelings of me as a child, as a baby, not me as an adult… And what I have experienced over and over again, is such a deep loneliness, which I cannot explain rationally, because I do have a large social network, I am well integrated. But nevertheless this loneliness still creeps up on me from time to time, (…) It's like I am catapulted back into another state. Into an earlier one*. (Participant 1, age 31)

“*Yes, definitively. I have three sisters, for example. I mean, everybody can be different of course, but I am… the other three are so similar and I somehow am so different. Yes, this is why I believe it must come from being born a preterm.”* (Participant 5, age 53).

### Health Care

In the short questionnaire preceding the actual interview 70% of the participants said that overall, they were satisfied with their current health care provision.

One third (30%) of the participants indicated that they were seeking professional health care regularly, 70% said that they only visit a health care provider on demand or in case of acute illness. In all of the participants the health care provider primarily involved was the general practitioner or family doctor. Depending on their health care status, healthcare from other specialists was sought. When asked how satisfied participants were with regards to their status as former preterms, only 55% of the participants indicated that they were satisfied with their current health care. The majority of participants (80%) claimed that their prematurity is never mentioned by their current health care providers and that their status as adults born preterm does not influence current treatment or counseling options by health care professionals. As a result, only 40% of the participants feel well-informed and advised about the potential risks associated with preterm birth.

“*(.) that the topic of prematurity played a role until I was maybe 15, 16 years of age (.) meaning that doctors actively asked about being born preterm or something and actually now, in adult life, it is never mentioned again.”* (Participant 14, age 27)

Half of the participants who reported to regularly seek medical treatment said that they were unsatisfied with their current health care. In comparison, of those participants who reported to seek medical treatment only on demand or in case of acute illness only 14% said that they were unsatisfied with health care services. Perceived reasons for not being satisfied with health care were:

a) lack of consideration for their prematurity status by current health care providers.
b) unmet need for specialized health care services with regard to their former prematurity.

Some ABP reported difficulties in receiving adequate treatment targeted to their complex medical conditions. Perceived barriers for receiving adequate treatment were again lack consideration and lack of knowledge about the long-term effects of prematurity by current health care providers as well as the small time-windows health care providers have for treating the individual patient, which impedes them from carefully listening to their patients' complaints and from advising them appropriately.

Another problem described were rejected reimbursements by health care insurance companies, an example being the rejected continuation of physiotherapy for an ABP with cerebral palsy.

The perception that current health care providers do not sufficiently consider prematurity status was independent of the participants' education level. The proportion of participants stating that prematurity is considered by their current health care provider was equal between those holding a high school diploma corresponding to University entrance level and those holding lower school diplomas (50%, respectively).

The older a patient gets the less likely it is that her or his current health care provider informs or counsels her/him with regard to potential risks of former prematurity. More than half of ABP >30 years indicated that their health care provider rarely or never speaks with them about potential long-term risks of former prematurity whereas in the age group of ABP <30 years the majority of the participants indicated that their health care provider *does* inform them about potential risks of former prematurity.

Consequently, older ABP expressed more worries or fears concerning potential long-term sequelae of their former prematurity than younger ABP (33 vs. 18%) and the dissatisfaction with health care services regarding their former prematurity was greater among older ABP >30 years of age.

“*The regular doctors, no (they do not consider my former prematurity), not really. But it definitively is a topic with my healer and the osteopath, obviously. So it is more of a topic in alternative medicine.”* (Participant 10, age 39)

“*I once went to see a pulmonologist, but he was like: “Nope, everything is fine.” He did not take me seriously, he acted as if I was a bit of hypochondriac who is unnecessarily still sitting in his waiting room. This may be a personal opinion, but I feel that there is not much knowledge (on the topic of former prematurity) around.”* (Participant 1, age 31)

“*Well yes, I would have wished – at least concerning the cerebral palsy – that someone would have told me about how it develops with age. Because nobody is talking about this. (…) I practically had to google all the information myself.”* (Participant 16, age 26)

Prematurity status is more often considered by current health care providers in participants with impaired physical health compared to participants who rarely or never suffer from physical health impairments.

“*(.) that the topic of prematurity played a role until I was maybe 15, 16 years of age (.) This may also be due to the fact that fortunately you can't see anything from the outside and that I don't have any obvious impairments.”* (Participant 14, age 27)

Of the participants who often or always suffer from mental health sequelae only one third felt that their prematurity status is adequately considered by their current health care providers, whereas the majority stated that their prematurity status rarely or never plays a role in their provider's treatment or counseling approach.

“*(…) I have had rather negative experiences. It (my prematurity) has been mentioned once in psychotherapy but it was never discussed in depth or taken serious. And my GP said, I was welcome to show her relevant scientific studies if I was interested in the topic but did not address my concerns at all.”* (Participant 6, age 54).

### Social/Attachment Behavior

A substantial proportion of ABP expressed the perception that their former prematurity negatively influences their social life and their relationships as adults.

There is a difference regarding the perceived influence of prematurity on social life and attachment behavior between the age groups of ABP <30 years of age and ABPs >30 years of age. In the older ABP group over half of the participants (56%) perceived that former prematurity negatively influences their social life, in the younger age group this was of concern in about one third of the participants (36%).

75% of participants who often or always suffer from physical health sequelae attributed to their former prematurity reported a negative influence of former prematurity on their social/attachment behavior. Physical limitations resulting from preterm birth sequelae were perceived as barriers for the maintenance of a satisfactory social life. Especially ABP suffering from cerebral palsy described that their medical conditions are likely to hold them back from participating in social activities, often causing a feeling of loneliness.

“*No, (I can) definitely not (participate in activities with friends without limitations). And often, people have to change plans on behalf of me and I feel very uncomfortable about that.”* (Participant 16, age 26)

“*Well, most of the time it is possible for me, but as soon as it goes into the direction of sports activities then it becomes more difficult. For example, if they go skiing at the weekend in the winter, that is not possible. If they go to the high wire garden, that is not possible.”* (Participant 15, age 30)

The perception that former prematurity negatively influences their social/attachment behavior was far less prevalent in participants who reported rare or no physical health sequelae (27%). Participants who suffer from mental health sequelae attributed to their former prematurity were most likely to report a negative influence of former prematurity on their social/attachment behavior (78%) Here, introversion, difficulties in approaching people and difficulties in making friends were named as main barriers.

At the time of our interview, only 3 (15%) of the ABPs were in a stable relationship 12 (60%) participants stated that they had been in a relationship before, 2 (10%) participants were divorced, 2 (10%) had never been in a relationship and one participant declined to talk about the issue. Romantic relationships of most ABP were described as emotionally difficult and demanding. Difficulties in permitting closeness as well as a strong sense of dependency were described. Two participants described a lack of enjoyment in physical contact and sexual activity as problematic for their relationships. None of the interviewed participants had children and the subject of parenthood was burdened with fears. Participants expressed concerns about the possibility that their own child could also become a preterm baby and possibly inherit unfavorable mental predispositions.

“*I totally like children, but to be honest, I don't think I want children just because I'm afraid that my child - it is said that the physical impairments won't get passed on because they are due to oxygen deficiency, so there I cannot transmit much - but I'm afraid that my psychological self will be passed on. And I don't want that.”* (Participant 11, age 33).

### Education and Professional Life

When asked if they had (yet) reached their professional goals, 55% of all participants answered that yes, they had achieved their professional goals.

Of the participants who reported to suffer from physical health sequelae attributed to their former prematurity half said that they had reached their professional goals. Reported barriers were physical limitations due to long-term sequelae of pre-mature birth. One participant with cerebral palsy for example, cannot exercise her dream profession of being a kindergarten teacher, as this would be physically too demanding for her. Another participant is in early retirement because she was not able to cope with the burden of regular working schedules. She is very grateful for not having to exceed her limits, but expressed her wish to be more resilient. Of the participants who reported rare or no physical health sequelae, 64% said that they had reached their professional goals. Barriers to pursuing their careers and to reaching their professional goals were difficulties in finding an adequate training position, unemployment and uncertainty about having chosen the right occupation.

Of participants who reported to suffer from mental health sequelae attributed to their former prematurity, substantially less, only 33%, said that they had reached their professional goals. Reported barriers to achieving their professional goals were the perception of a too high workload and the uncertainty about having chosen the right occupation.

During the interview all participants were asked: “Given with what you know now, if there was a specialized custom-tailored health care service for adults born preterm, would you be utilizing this service?”

All of the participants (100%) said yes, that they would utilize a specialized health care service for adults born preterm.

Expected contents and benefits expressed for such a service are listed in Table 6.

**Table 6:** Expected contents and benefits of specialized custom-tailored health care services for ABP.

**Expected contents and benefits of specialized custom-tailored health care services for adults born preterm**
Improving information and counseling for the individual affected ABP
Raising awareness for the topic of ABP among other health care providers and the public
Concrete support with applications and referrals (specific health care services, aides and appliances) for ABP
Adequate time slots for individualized counseling of every ABP patient
Interdisciplinary prevention and treatment approach for ABP
Specialized knowledge and experience with the specific patient population of ABP
Possibility of exchange with other affected ABP

Participants also mentioned the importance of accessibility for affected individuals impaired to travel who would still be interested in gathering information and provider's guidance from a respective health care service tailored to the needs of adults born preterm. This could potentially involve a website where questions can be posted and discussed, email and/or phone contact with the provider.

## Discussion

A large body of literature exists on the medical and mental health outcomes of very pre-mature infants in early adulthood. Most studies suggest that a significant proportion of former preterm infants have made a reasonably successful transition into adulthood, even though some differences exist when compared to adults born at term (36–39). However, to date, hardly any studies report on the *personal* perspectives of former pre-mature infants and none, to our knowledge, address the question of how former preterms feel embedded in established health care structures or if they face any health care-related barriers (26).

This qualitative study focused on a sample of adults born very preterm, who had not been in any kind of systematic follow-up program focusing on the trajectories of their prematurity. We aimed to understand how these ABP themselves evaluate their actual health status and to learn about their health care experiences as adults.

One overarching theme emerged during our data analysis. It is the participants' perception of *the invisibility of and the unawareness for their condition (of being a former preterm)*. This theme strongly affects various domains of life, including not only personal relationships and professional development but also participants' attitudes toward health and health care.

The majority of ABP in our sample perceived that adult health care providers are largely unaware of their patients' prematurity status and of potential long-term sequelae of prematurity. They felt that many, especially more subtle health impairments which they attributed to their former prematurity remain unperceived: low basal energy level, low stress tolerance, hypersensitivity to external stimuli and a higher effort to reach and maintain the same level of performance as peers.

The core statement is that former prematurity is not considered (a problem) in current health care.

The reported diagnoses, including ICD-10-codeable diseases (40), as well as the self-perceived health impairments concerning participants' fitness and performance capability are in line with the results of former longitudinal and health-related quality of life studies which describe impairments in ABP's physical and mental health state (39, 41–47). The prevalence of physical and mental health sequelae in our sample was relatively high, which may reflect a recruitment bias, as the majority of participants were recruited with the help of patient and parent advocacy groups and therefore may comprise more individuals with a high subjective or objective burden of disease.

Most participants' expressed anxiousness toward founding their own family or becoming parents themselves. As reasons they named fear of their children being born pre-mature as well and fear of passing on unfavorable mental health attributes to their offspring. Of notice, large Scandinavian register studies have shown that individuals born preterm or with low birthweight were less likely to ever be in a registered partnership or to become parents (39, 48). Other authors have also found that ABP, with or without impairments, were more likely to be single compared to term born peers (27) and that adults born preterm or with low birthweight were less likely to experience a romantic partnership, sexual intercourse, or parenthood than their peers who were born full-term (49).

Multiple participants found it challenging to pursue their educational goals and felt that they were unable to perform professionally as well as others in their age group. This is important as various studies have shown that ABP are less likely to achieve higher education qualifications and to be employed as adults than are their term born peers, leading to adverse impacts on their socio-economic outcomes (46).

Our data suggest that the self-perception of ABP changes as they age.

Older ABP perceive to have more mental health sequelae which they attribute to their former prematurity than younger ABP. On the socio-emotional level, older participants reported to experience more difficulties in their social/attachment behavior than younger ABP. This may be due to the fact that, overall, socialization and maintenance of friendships become harder as individuals age. Also, all but one participant of the older ABP (age group >30) in our sample were single at the time of interview, which may augment the risk for subjective and objective mental health impairments. These findings are in line with other studies which have found that at young adulthood ABP express a relatively high level of self-perceived health-related quality of life, (27, 50, 51) which can substantially differ from their environments' perceptions. (52) As they reach their fourth, fifth, or sixth decade of life, this positive self-perception may change as new health-related and social challenges are faced and formerly unconsidered concerns appear. Interestingly, a recent Canadian cohort study found that older ABP (i.e., ABP in their 4th decade of life) were less likely to be married or to have children of their own in comparison with term born peers, whereas no differences in these domains were found between younger ABP and those born at term (27).

Also, the dissatisfaction with health care services regarding their former prematurity status and potential long-term sequelae is more pronounced in older ABP. In contrast to ABP's perspectives it seems that the further in the past birth and perinatal circumstances lie, the less consideration do they get from adult health care providers. These are conflicting findings that could be problematic as ABP are known to be more susceptible for age-associated diseases, may earlier exhibit risk factors for certain pathologic conditions such as cardiovascular disease and should therefore be monitored more intensively as they get older. (12, 23)

Participants perceive knowledge gaps–on the patients' as well as on the providers' side–and express a strong desire for information and counseling on the relevant health topics for adults born preterm. Importantly, all participants of our sample claimed that they were interested in utilizing a special, custom-tailored and individualized health care service, putting today's health issues into the larger frame of their perinatal history.

Our interview data show that there is at least a subgroup of ABP “out there,” who suffer from the underperception of being a former preterm with specific needs and elevated health risks. Considering the expected long-term sequelae of prematurity these individuals point toward a blind spot in the German health care system: adequate health services for ABP, after their transition from child- into adulthood.

The availability of custom-tailored ABP health care services may provide an institutionalized opportunity for ABP to access health information, health care prevention and counseling with a focus on their individual risk profiles. This, on the provider's side, should imply a more “holistic” medical approach which treats the ABP as an individual with an elevated but variable risk profile rather than focusing on specific symptoms or diagnoses.

From a public health perspective the pooling of ABP at an institutionalized service will help to collect a lot of valuable, prospective health data and thus increase our knowledge on the long-term effects of former prematurity. Furthermore, adequate health prevention may help to detect the development of diseases of later adult life in time, when preventive measures are still applicable. This may eventually help to reduce health care related costs for this steadily increasing and aging patient population.

Our pilot study has several limitations. As participants were mainly recruited via the websites and contacts of two patient and parent advocacy groups, recruitment bias is a major limiting factor. In this context one has to be very careful when interpreting causalities. Prematurity may be interpreted by participants as the cause for symptoms that are in fact unrelated to preterm birth or of multifactorial origin. Future research on the topic should aim to perform random recruitment in order to achieve higher representativeness of study results. Furthermore, a desirability bias implying that the study participants anticipated what the interviewer expected to hear from them, cannot be ruled out. Another selection bias is possibly caused by the fact that the majority of our study sample comprised participants with a high educational level. As neurocognitive and behavioral dysfunctions are more prevalent in very small preterm infants (45, 53), the overall high educational level of our sample is surprising and not representative for the very low birthweight patient population in general (46). With 70% of study participants holding a highschool diploma the quota is higher than in the general German population (34, 35).

Our study comprises individuals embedded into the German health care system only. ABP's perceptions with access to other national health care systems may differ from the ones of our German participants. Furthermore, differences between ABP living in the countryside in contrast to ABP living in urban areas may be prevalent.

Finally, our study results only reflect participants' personal perspectives on health care providers' practice and do not comprise the providers' side of view. Future research should aim to assess in how far the status of former prematurity is considered among adult health care providers in their everyday practice.

## Conclusion

Adults born preterm are a steadily increasing patient population. There seems to be at least a subgroup of ABP who suffer from health impairments related to their former prematurity and who perceive that they have no designated health care structures to turn to. The German healthcare system has yet to recognize ABP as a specific at-risk-population, who may require special services in order to enable effective prevention and to meet ABPs individual health-associated needs. In order to fully understand the impact of very preterm birth and respective health care needs in adulthood we should continue to include the perspectives of individuals born preterm in the process of research and in the development of appropriate guidelines.

## Data Availability Statement

The raw data supporting the conclusions of this article will be made available by the authors, without undue reservation.

## Ethics Statement

The studies involving human participants were reviewed and approved by Ethical Committee of the Hamburg Medical Association. The patients/participants provided their written informed consent to participate in this study.

## Author Contributions

AP and LT contributed equally to the design of the study, development of the interview guide, transcription and analysis of the data, and preparation of the manuscript. DL contributed to the development of the interview guide, gave methodological advice, supervised data collection, and contributed to data analyses. CE edited scientific English and critically reviewed and revised the manuscript. OK contributed to the conception and design of the study idea and provided professional guidance to the raters throughout the analytic process and revised the manuscript critically for important intellectual content. DS supervised the whole study group, gave advise for the conception and design of the study as well as for the analyses, critically reviewed and revised the manuscript, and approved the final manuscript as submitted. All authors contributed to the article and approved the submitted version.

## Conflict of Interest

The authors declare that the research was conducted in the absence of any commercial or financial relationships that could be construed as a potential conflict of interest.
