# Supplementary material for: Lost in Transition: Health Care Experiences of Adults Born Very Preterm—A Qualitative Approach
Source: Front Public Health. 2020 Nov 30;8:605149. doi: 10.3389/fpubh.2020.605149 (PMC7793891; doi:10.3389/fpubh.2020.605149)
Supplement: Supplementary file 1 [file Data_Sheet_1.pdf]

Analytic code-system

**Deductive codes**<br>(quantity of code assignments)	**Inductive subcodes**<br>(quantity of code assignments)
**1. role of preterm birth (PB) today (82)**	1.1 PB is a mental issue (17)<br>1.2 PB is a physical issue (35)<br>1.3 PB is of no issue (1)<br>1.4 other occupation with PB (7)
**2. long-term effects of PB** (55)	2.1 uncertainty about causalities (32)<br>2.2 late and long-term effects of PB (22)<br>2.2.1 always been an issue (3)<br>2.2.2 recently became an issue (8)<br>2.2.3 never been an issue (7)
**3. concerns regarding own PB** (20)	3.1 fear of PB in own offspring (3)
**4. physical health** (306)	
4.1 clinical diagnoses (112)	4.1.1 speech disorders (6)<br>4.1.1.1 speech impairment in childhood (1)<br>4.1.1.2 stuttering (5)<br>4.1.2 ophtalmologic diseases (16)<br>4.1.2.1 myopia (4)<br>4.1.2.2 strabism (4)<br>4.1.3 hearing loss (3)<br>4.1.4 reduced fine motor skills (9)<br>4.1.5 impaired balance (3)<br>4.1.6 infantile cerebral palsy (34)<br>4.1.6.1 spasticity (27)<br>4.1.7 pulmonary disease (6)<br>4.1.7.1 asthma bronchiale (3)<br>4.1.7.2 reduced lung capacity (2)<br>4.1.8 hydrocephalus (1)<br>4.1.9 other disease (22)<br>4.1.10 no chronic disease (12)
4.2 perception of own health (52)	4.2.1 sleep disturbance (3)<br>4.2.2 feeling unconnected to own body (2)<br>4.2.3 low basal energy level (29)

	4.2.4 prolonged recovery after illness (2)<br>4.2.5 susceptibility to disease (8)<br>4.2.6 impaired sense of orientation (7)
4.3 physical performance (13)	
4.4 medical treatment (19)	4.4.1 treatment only on demand (13)<br>4.4.2 regular treatment (6)
4.5 health care seeking behaviour (22)	
4.6 PB considered by health care provider (25)	
4.7 use of complementary medicine (28)	
4.8 preventive health care (34)	4.8.1 prevention independent from PB (11)<br>4.8.2 special prevention due to PB (4)
**5. mental health** (236)	
5.1 mental health diagnoses (27)	5.1.1 burnout (2)<br>5.1.2 depression (21)<br>5.1.3 anxiety disorder (1)<br>5.1.4 regulation disorder (2)<br>5.1.5 trauma (1)
5.2 mental characteristics (100)	5.2.1 lack of self-confidence (3)<br>5.2.2 difficulty to concentrate (4)<br>5.2.3 emotional hypersensitivity (5)<br>5.2.4 introversion (8)<br>5.2.5 attachment disorder (5)<br>5.2.6 reduced filtering capacity for external stimuli (11)<br>5.2.7 feeling of loneliness (7)<br>5.2.8 need for control (6)<br>5.2.9 susceptibility to stress (8)<br>5.2.10 anxiety (15)<br>5.2.11 restlessness (1)

	5.2.12 feeling immature (3)<br>5.2.13 stubbornness (1)<br>5.2.14 autistic traits (17)<br>5.2.15 developmental delay (4)<br>5.2.16 memory impairment (2)
5.3 psychotherapy (49)	5.3.1 currently undergoing psychotherapy (19)<br>5.3.2 formerly undergone psychotherapy (7)<br>5.3.3 contemplating to begin psychotherapy (5)<br>5.3.4 no psychotherapy (5)<br>5.3.5 consideration of PB in psychotherapy (8)<br>5.3.6 therapist's expertise on PB (4)
5.4 self-perceived influence of PB on the mental health (22)	5.4.1 PB influences mental health (18)<br>5.4.2 PB does not influence mental health (4)
5.5 challenges in everyday life (17)	
5.6 mental resilience (20)	5.6.1 higher than peers (1)<br>5.6.2 same as peers (7)<br>5.6.3 lower than peers (11)
**6. health care** (68)	
6.1 evaluation of own health care (19)	
6.2 unmet expectations from insurance company and health care system (21)	
6.3 custom-tailored health care service for ABP (27)	
**7. education and profession** (227)	
7.1 education (14)	

7.2 studying / higher degrees (14)	
7.3 school time (35)	7.3.1 difficulties at school (48)<br>7.3.1.1 difficulty to concentrate (2)<br>7.3.1.2 physical impairment (10)<br>7.3.1.3 mobbing (14)<br>7.3.1.4 learning difficulties (10)<br>7.3.1.5 school as stress factor/burden (8)
7.4 support and therapeutic interventions (24)	
7.5 profession (49)	7.5.1 work as stress factor/burden (15)
7.6 professional achievement (21)	
7.7 requests for educational/ professional support (21)	
**8. family** (156)	
8.1 family constellation (44)	8.1.1 family conflict (16)<br>8.1.1.1 domestic violence (6)
8.2 other preterm family members (19)	
8.3 role of PB within the family (41)	8.3.1 feeling of not matching the family (5)<br>8.3.2 PB as a family trauma (6)<br>8.3.2.1 PB unmentioned within the family (3)
8.4 being treated differently due to PB (51)	8.4.1 positive evaluation (4)<br>8.4.2 negative evaluation (6)<br>8.4.3 overprotected (10)<br>8.4.4 same treatment as others (7)
**9. living situation** (21)	

**10. partnership** (127)	
10.1 romantic relationship (39)	10.1.1 currently (3)<br>10.1.2 formerly (27)<br>10.1.3 divorced marriage (4)<br>10.1.4 never (2)
10.2 difficulty finding a partner (11)	10.2.1 too high expectations (2)<br>10.2.2 too much else to do (1)<br>10.2.3 difficulty in approaching people (3)<br>10.2.4 missing occasions (3)<br>10.2.5 similarity of potential partners to negative traits of parents (2)
10.3 wish to be in a romantic relationship (9)	10.3.1 yes (4)<br>10.3.2 not anymore (3)<br>10.3.3 desire for physical intimacy (2)
10.4 self-perceived problems in romantic partnerships (30)	10.4.1 being too possessive (3)<br>10.4.2 experiencing partnerships differently from others (7)<br>10.4.3 inability to trust others (1)<br>10.4.4 dislike of physical intimacy (2)<br>10.4.5 inability to find a partner (3)<br>10.4.6 need of personal freedom (5)
10.5 having own children (19)	10.5.1 fear of PB to be repeated in own offspring (2)<br>10.5.2 fear of passing on unfavorable mental predispositions to offspring (2)
10.6 role of PB in partnership (17)	
**11. leisure time activities** (140)	
11.1 hobbies and activities (24)	
11.2 satisfaction with number/quality of friendships (44)	11.2.1 little need for social contacts (2)<br>11.2.2 difficulty in getting to know people (12)

	11.2.2.1 due to physical limitations (3)<br>11.2.2.2 inability to trust others (3)
11.3 participation in social activities (19)	
11.4 long-standing friendships (15)	
11.5 role of PB in friendships (17)	
11.6 role of PB in everyday social life (20)	
